# The Effects of Motor Imagery on Pain in Lower Limb Sports Injuries: A Systematic Review and Meta-Analysis

**DOI:** 10.3390/healthcare10122545

**Published:** 2022-12-15

**Authors:** George Plakoutsis, Eleftherios Paraskevopoulos, Athanasios Zavvos, Maria Papandreou

**Affiliations:** 1 Department of Physiotherapy, University of West Attica, 12243 Athens, Greece; 2 School of Physical Education and Sport Science, National and Kapodistrian University of Athens, 11527 Athens, Greece

**Keywords:** motor imagery, sports injuries, lower limb sports injuries, sports psychology, sports rehabilitation

## Abstract

This review evaluated the efficacy of Motor Imagery intervention in athletes with lower limb sports injuries that could affect their pain levels during rehabilitation. We carried out a thorough research of the scientific literature for RCT studies in athletes with lower limb musculoskeletal sports injuries including search terms Motor Imagery AND pain, Motor Imagery AND sport injuries, Motor Imagery AND lower limb. We searched 3 major databases, PubMed, Scopus, and ScienceDirect, with the search period ranging from their inception until May 2022. We assessed the quality of the studies using the PEDro Scale and the data was recorded and extracted with the use of Mendeley software. The search criteria resulted in a pool of 10.107 possible articles. Upon completion of the selection procedure, only 3 RCT studies met the inclusion criteria with a total of 60 injured athletes (*n* = 18 with ankle sprain and *n* = 42 with ACL injuries). The meta-analysis showed no statistically significant positive effects of MI intervention on pain intensity after lower limb sports injuries (*n* = 60; MD = −1.57; 95% CI: −3.60 to 0.46; I^2^ = 50%; *p* = 0.13). The limited number of studies could justify the statistically insignificant effect of MI, but although the methodological quality of the studies was moderate to high, the heterogeneity of them was also relatively high. More RCT’s are required to explore the effect of MI on pain in athletes with lower limb injuries in order to address psychophysiological processes during rehabilitation.

## 1. Introduction

Sports injury is a prevalent incident that disrupts athletes’ careers and their overall biological, psychological, and social well-beings. Consequently, a great amount of research is engaged in identifying lower limb injury recovery procedures, thus promoting a healthy ‘return to play’ condition for athletes of different skill levels [1,2].

However, lower limb sports injuries such as on the ankle joint are considered to be one of the most frequent injuries during athletic activities accounting for 30% of all sports injuries [3]. Furthermore, research shows that one of the most common musculoskeletal sports traumas that accounts for 48 out of 1.000 patients are knee injuries with 9% suffering from anterior cruciate ligament (ACL) rupture [1] with lack of adequate rehabilitation process. Research also suggests that knee function, pain, and fear of re-injury play a role in athletes’ successful ‘return to play’ condition [1]. In fact, some athletes are facing symptoms of post-traumatic stress such as anxiety and high pain levels [4,5,6]. Many therapeutic modalities have established their efficacy in the rehabilitation process with respect to sports psychology. Psychological strategies including motor imagery (MI), preparatory arousal, self-talk, attentional focus, and goal-setting have been used over time with athletes, either for improving athletic performance or in the rehabilitation procedure [7,8,9,10,11]. 

Pain is considered one of the most depleting complications among musculoskeletal sports injuries [12,13]. It could lead to fatigue, anxiety, sleep disorders, and psycho-social disturbances [14,15]. Musculoskeletal pain is a vast health condition that requires consultation [13,16]. It is considered a major risk factor that inhibits rehabilitation, resulting in poor functional outcomes and increased disability [17]. Pain can be classified as acute, recurrent, and chronic (the latter usually lasts more than 3 months from pain onset) [13,14]. Moreover, it can be further classified into three categories that apply to all pathological conditions and sports injuries, namely nociceptive, inflammatory, and neuropathic pain [14,18]. Neuropathic pain can be divided into peripheral-involving the peripheral nervous system in pathologies, such as diabetes, and central-involving the central nervous system in pathologies, such as strokes and multiple sclerosis [14,18]. Pain is a complicated, unpleasant experience which affects both the cognitive and motivational spheres and, therefore, the behavior of those who suffer from it [19]. 

MI is progressively included in rehabilitation as an adjunct therapeutic modality for trauma or injury management [20,21,22]. In reality, MI has gradually become one of the most popular technique among athletes for both rehabilitation processes (e.g., pain reduction) and performance optimization purposes (e.g., self-efficacy, injury anxiety) [2,6]. MI is defined as a voluntary process of mental simulation and representation of movements without actual execution [2,23,24]. Furthermore, it is considered to be a progressive motor cognitive procedure of mental rehearsal [25,26] due to the activation of motor regions in the brain when lacking an actual overt movement [1,26]. MI can be performed through two definite visual methods, namely the internal or external perspective and kinesthetic imagery (refers to someone’s ability feeling the movement) [22,26]. The internal perspective concerns the mental performance of a motor task from within the body and the external perspective concerns the mental performance as if it is outside the body. The internal perspective has been shown to be the preferred choice of rehabilitation due to the high levels of cortical activation [22,26,27]. On the peripheral nervous system, MI can imitate responses of the autonomic nervous system (ANS), which usually take place during physical exercise, such as playing a sport or exercising for physical recovery. These responses include the increase of heart rate, respiratory rate, and consequently the blood oxygen saturation—SpO_2_ rate [23,28,29]. MI showed that it could normalize cortical representation, while reducing pain symptoms in patients with regional complex pain syndrome whereas the excessive cortical representation of the body parts responded positively with the intensity of pain. Therefore, the use of MI may be beneficial in athletes with lower limb musculoskeletal injuries [22]. However, the effects of MI on pain still remain unclear. Many studies have revealed the effectiveness of MI intervention in pain reduction [30,31], whereas other studies have shown no effect on pain levels [32,33].

While the effectiveness of MI in relation to sports performance is well-established, there is an insufficient amount of Randomized Controlled Trial studies focusing on lower limb sports injury rehabilitation [1,2,22,26,34,35] and its effect on athletes’ recovery period. Focusing on psychological strategies such as MI may also improve injury prevention and rehabilitation strategies. Additionally, it seems reasonable to study the effect of MI on pain in athletes with lower limb musculoskeletal sport injuries. Therefore, evaluating MI effects in lower limb sports injuries is extremely important for all clinicians in order to establish new therapeutic strategies both in terms of injury management and ‘return to play’, based on bio-psychological factors. Thus, the aim of this systematic review was to evaluate whether the MI intervention in athletes with lower limb sport injuries could affect their pain levels during rehabilitation.

## 2. Materials and Methods

### 2.1. Eligibility Criteria

Studies that were selected for further critical appraisal met the following criteria determined a priori: (a) randomized controlled trials (RCTs), (b) including only athletes with no limitations on types of sports activities, or of athletic level, or age and gender, (c) investigated lower limb sports injuries (acute or overuse) of musculoskeletal etiology with no limitation in types of injury, (d) injured athletes who still receiving some form of treatment, (e) lower limb injury was diagnosed by a clinical professional, (f) received MI as a treatment, (g) compared the Motor Imagery intervention, both visual (internal or external) and kinesthetic, (h) between other interventions, e.g., conventional physical therapy, placebo Motor Imagery, relaxation techniques, medical treatment, and (i) investigated the assessment outcome of pain (acute, recurrent, and chronic), and how it was affected from the MI intervention compared to other therapeutic modalities. The exclusion criteria included in this study determined a priori were: (a) interventions other than Motor Imagery (e.g., Mirror Therapy), (b) sports injuries to the rest of the body except the lower limb (e.g., head, upper limb and spinal injuries), (c) healthy individuals, (d) observational and cross-sectional studies, case studies and case study series, and e) outcome measures other than pain (e.g., balance, range of motion).

### 2.2. Search Strategy

A systematic search of the research literature was administered for RCTs studying the effects of MI on pain after lower limb musculoskeletal sports injuries. Studies in English were found through electronic databases PubMed, Scopus, and ScienceDirect with the search period ranging from their inception until May 2022. The following keywords were used to search for terms: motor imagery AND pain, motor imagery AND sports injuries, motor imagery AND lower limb.

### 2.3. Eligibility Assessment 

The search was conducted by 2 reviewers (GP and LP). Articles were exported to Mendeley software and duplicates were removed [36]. The 2 reviewers independently screened and assessed the titles and abstracts of the remaining articles. The identification of relevant studies was achieved by screening the full text of each article based on the inclusion and exclusion criteria established a priori. Any discrepancies between the 2 reviewers were resolved with the help of a 3rd reviewer (MP).

### 2.4. Outcome Measures

The outcome that was examined in this study was pain intensity in order to provide results of the MI effects in sports injuries. Pain intensity is usually assessed using the visual analogue scale (VAS) or numerical rating scale (NRS). These tools are valid and reliable for measuring pain intensity [37]. Moreover, there are many other tools for pain assessment such as the brief pain inventory and the pain quality assessment scale which have also been proven valid and reliable [15]. In our study, VAS and NRS were used for meta-analysis. Nevertheless, studies which have used other assessment tools for pain were also included.

### 2.5. Data Extraction and Quality Assessment

The methodological quality of the studies was assessed using the PEDro Scale [38]. The PEDro scale consists of 11 criteria: specified eligibility criteria, random allocation, concealed allocation, similarity of the baseline characteristics of the participants, blinding of the participants, therapists and assessors, measurements of at least one key outcome from more than 85% of the participants, statistical analysis, comparison between groups, and measures of variability. Two independent reviewers (GP and LP) assessed all studies. Quality assessment was performed for all studies, including those not published in peer-reviewed journals (e.g., dissertations) thus meeting the exclusion criteria. Any disagreement between the reviewers regarding the data extraction was resolved in discussion between the three reviewers (GP, LP, MP). There was no unresolved issue between the reviewers. The data was recorded and extracted with the use of Mendeley software.

### 2.6. Analysis

A meta-analysis was conducted regarding the effects of MI intervention on the outcome (pain intensity level). We calculated the mean difference (MD) with 95% confidence interval (CI) for each trial and also the overall MD with 95% CI. In order to quantify the heterogeneity, we used the I^2^ statistic for all comparisons and the random effects models if the heterogeneity was high. 

All the included studies showed moderate to high methodological quality (PEDro Scale scores > 7 points) which ensured the sensitivity of the results. For all analysis, we used the software RevMan 5.

## 3. Results

### 3.1. Study Selection

**Figure 1:**
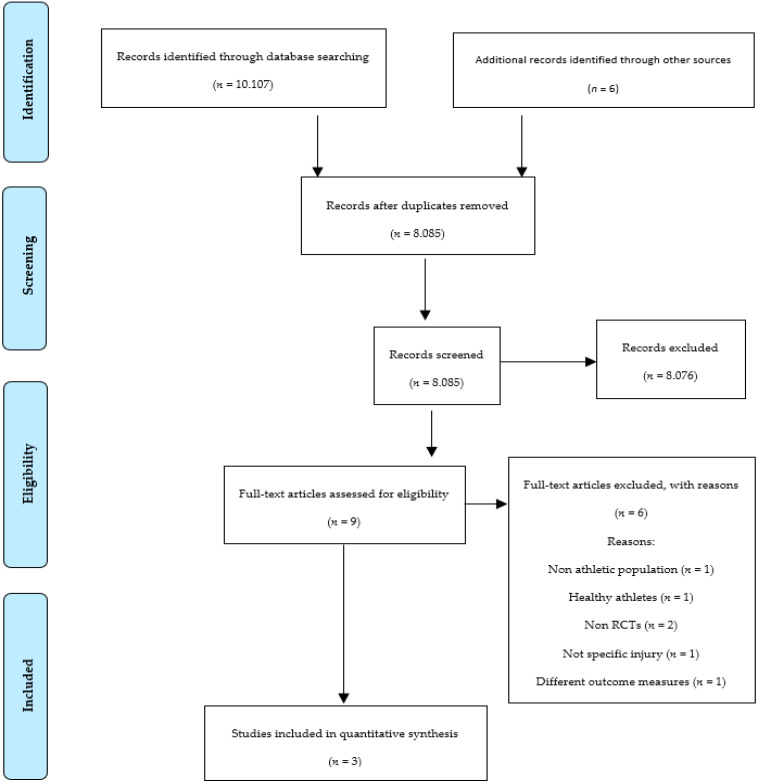
PRISMA flow diagram depicting the search procedure.

The search strategies resulted in an exploratory pool of 10.107 possible articles (Figure 1). Upon completion of the selection procedure, only three studies met the inclusion criteria. Nine studies examined the effects of MI after a lower limb sports injury. However, one of them assessed the non-athletic population [39], one assessed healthy athletes, two were not RCTs [40,41], one did not include information regarding the injured structure [42], and one examined different outcome measures [43]. Thus, three studies investigated the effects of MI on pain after a lower limb sports injury and are included in this review. Each of these studies was conducted considering characteristics such as participants’ age, gender, athletic level, MI or other relaxation techniques, and the type of lower limb injury (Figure 1).

### 3.2. Study Characteristics

The number of participants in each study were aged between 18 to 50 years old, including both males and females. The athletic level of the participants varied, thus including both amateur and competitive athletes from different sports. Overall, the number of participants included in this review was sixty injured athletes with a lower limb sports injury (ACL injury or ankle sprain). 

Many methodological settings of MI protocol and mode of exercises (number of weeks, sessions, duration, images and relaxation techniques or instructions) were presented in these studies. Christakou and Zervas [44], implemented Relaxation and Imagery intervention for twelve sessions in four weeks, with a duration of forty-five minutes. Participants were randomly allocated in two groups: one experimental and one control group (physical therapy), respectively. Injured athletes had a grade II ankle sprain, came from a variety of sports and training levels and were aged 18–30. Cupal and Brewer [45], applied 10 sessions of Relaxation and Guided Imagery every second week over a period of six months with a duration of 10–15 min in athletes aged 18–50 with ACL injuries from different sports and training levels (competitive and amateur). Participants were randomly divided into three groups: relaxation and guided imagery, placebo (attention, encouragement, and support) and control group (physical therapy), respectively. Lastly, Lebon, Guillot, and Collet [46], applied twelve sessions of Kinesthetic Imagery every two days for five weeks with a duration of 15 min in 12 athletes with ACL injuries where 9 of them had competitive activity in various sports. Participants (aged 18–40) were randomly allocated into two groups: Kinesthetic Imagery and control group (physical therapy), respectively (Table 1).

### 3.3. Methodological Quality

The methodological quality scoring of the selected studies on the PEDro Scale varied between 7 and 9 with a median of 8 points. Overall, 2 of the included studies were rated with 7 points and 1 with 9 points which showed moderate to high methodological quality. There was no blinded procedure in the methodology of any of the studies, therefore the 9 points was the highest score (Table 2).

### 3.4. Effects of Intervention on Pain

**Figure 2:**
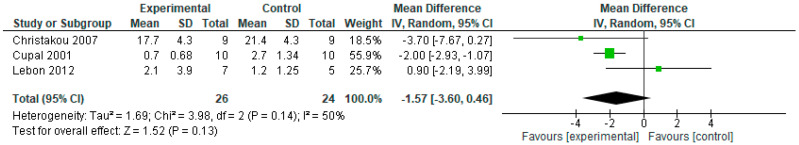
Analysis of MI intervention for pain intensity.

Pain was evaluated using the VAS in 2 studies [45,46] and the NRS in 1 study [45]. Hence, the study of Cupal and Brewer [45] showed a statistically significant reduction in pain intensity in the MI group compared to the placebo and the control group. On the contrary, the studies of Christakou and Zervas [44] and Lebon et al. [46] did not show a significant difference in pain intensity after MI intervention. The meta-analysis showed no statistically significant positive effects of MI intervention on pain intensity after lower limb sports injuries (*n* = 60; MD = −1.57; 95% CI: −3.60 to 0.46; I^2^ = 50%; *p* = 0.13), indicating no statistical difference and moderate to high heterogeneity. The results of this meta-analysis are based on low quality evidence, (Figure 2, Table 3). 

## 4. Discussion

The aim of this systematic review was to evaluate whether the MI intervention technique in athletes with lower limb sports injuries could affect pain intensity during rehabilitation thus reaching the ‘return to play’ period faster. No significant effects on pain intensity were discovered in the 3 studies analyzed. The lack of statistically reliable effects was firstly derived from the limited number of RCTs and their heterogeneity and, secondly, from the small sample size of the studies. Nevertheless, a strong positive effect was observed on pain intensity in one study [37] which is consistent with other studies assessing different populations and pathologies [15]. These findings suggest that MI might have a complementary therapeutic impact on pain intensity in athletes with lower limb sport injuries.

A limited number of studies have examined the use of MI on pain management in athletes with lower limb sports injuries. In spite of these limitations, the present systematic review highlights some interesting findings. This is the first systematic review to focus on the efficacy of MI interventions on pain, specifically in athletes with lower limb sports injuries. Despite the non-significant effects observed, there is a considerable amount of studies that examined the use of MI in treating pain. However, the results of these studies focused on different subject allocation criteria (healthy individuals, neurological pathologies, etc.), thus presenting fluctuations regarding the efficacy of MI [18,47,48,49,50]. Previous research supported the evidence that pain was the superior cause of psychological establishment in the Central Nervous System (CNS) [22]. MI could activate brain neural circuits that could also involve the neurons of perceived pain in a complex manner. MI is considered to be an effective cognitive therapeutic modality mostly in athletes and in the elderly with neurological disorders (e.g., stroke), [51,52] hence altering psycho-physiological responses which tend to have a positive contribution on perceived pain intensity.

However, our findings indicate that the use of MI could reduce pain intensity compared to cases when it is not used. Cupal and Brewer [45] showed a statistical reduction on pain perception when applying Relaxation and Guided Imagery in the experimental group, in comparison to the placebo group (attention, encouragement, and support) and the control group which followed their physical therapy programme only. On the contrary, Christakou and Zervas [44] did not show statistically significant results on perceived pain intensity in the experimental group which followed Relaxation Imagery in comparison to the control group that followed physical therapy. Additionally, Lebon et al. [46], did not show evidence of the effectiveness of MI on pain reduction in the experimental group which followed Kinesthetic Imagery in comparison to the control group which followed physical therapy. These findings indicate the conflicting results of the MI therapeutic effect. We suggest that this could be attributed to the fact that the MI interventions were focused on mental representation of movements (Kinesthetic Imagery) and secondly on pain management.

The study conducted by Cupal and Brewer [45] at their six month follow-up showed a significant difference in pain levels in the experimental group compared to the control group, whereas the studies conducted by Christakou and Zervas [44] and Lebon et al. [46] did not apply an evaluation on pain intensity in the long-term. This outcome might suggest that the intervention of MI after a surgery does not result in a short-term improvement on pain intensity and is therefore no faster than conventional physiotherapy. The healing process of injury and the timing of MI intervention could be another factor that influences its effectiveness.

Moreover, the duration of MI treatment differed between the methodological procedures of the studies [35,36,37]. Cupal and Brewer [45] reported that each MI session lasted from 10 to 15 min, and whereas Lebon et al. [46] reported that MI sessions lasted 15 min. On the contrary Christakou and Zervas [44] reported that MI protocol lasted 45 min. These mixed MI therapeutic regime modes are not agreeable to Dickstein and Deutsch [53] who found a negative relationship between the effectiveness of MI and its duration. They proposed that the optimal duration of every MI session was up to 20 min for healthy individuals whereas they suggested an MI session of only up to 10–15 min for individuals with neurological complications (e.g., stroke). 

Furthermore, many studies have shown that the brain shares the same representation of content and location between motor imagery and awareness in patients who suffer from chronic pain [14]. Pain is always associated with negative images and feelings, especially in athletes, thus affecting the healing process and ‘return to play’ criteria [54,55]. Additionally, MI could affect pain intensity by developing the activity of the motor cortex which is associated with pain perception [56]. Therefore, the type and the intensity of sports injuries could change the athletes’ response in MI intervention with regard to pain intensity. In our study, athletes derived from different competitive levels and different sports. This heterogeneity could have altered the MI effect on pain intensity. Cupal and Brewer [45] and Lebon et al. [46] used both amateur and competitive athletes in their studies, but the latter were a smaller sample. This could justify the inability to demonstrate the effectiveness of MI on pain intensity. On the contrary, Christakou and Zervas [44] used only amateur athletes in their study which could be an aspect that has influenced the outcomes. MI results in differing neurocognitive effects based on the level of expertise of the athlete. The athletic level and the athletes’ previous injuries could be factors that crucially need further investigation from sports psychologists and physiotherapists in order to understand the complexity of MI and its effect on pain [57]. Future evaluation of MI could include an assessment of known psychological variables such as depression, anxiety, self-confidence issues, and fear of re-injury. Further predictions could be made on the athletic population and how MI could influence pain intensity after lower limb sports injuries. 

MI involves the creation of mental images and the control of visualization. It has been successfully used in different pathologies such as cancer [47], musculoskeletal pathologies, and post-operative conditions [21]. Another indicator of the efficacious effect of MI on pain intensity is the pain gate theory, which dictates that, if the route of the painful stimulation could be altered by a pleasant stimulus, pain intensity levels could also decline [14]. The effectiveness of MI in cases where pain physiology is associated with peripheral factors (e.g., lower limb injuries) needs to be further investigated in order to correlate the known efficacious effect of MI on chronic pain with lower limb sports injuries [19]. Additionally, future studies should clarify that changes to the CNS due to MI implementation could influence the pain gate theory and investigate the neurophysiological mechanism that underlies the reduction of pain intensity. 

In accordance with the aforementioned studies, the efficacious therapeutic regime and mode of the MI technique on reducing pain perception needs to be further investigated in athletes with a lower limb injury. MI variations in moderators’ criteria need to follow a structured methodological approach with the same duration and frequency of sessions including instructions based on pain management.

### 4.1. Clinical Implications

This review provided a critical overview of 3 RCT studies based on the effectiveness of MI on pain intensity in athletes’ population with lower limb injuries using three major databases to identify the greatest number of applicable studies to the research question. The findings support the view that this is the only study focused on the effectiveness of MI specifically on the athletic population with lower limb sports injuries. The results showed that the MI intervention should be considered in the treatment of sports injuries combined with conventional physiotherapy. The advantage of MI is that it can be applied in cases where treatment is not possible due to immobilization or to the presence of psycho-social factors that do not allow the execution of movements. More research is needed in order to determine the best parameters of intervention for athletes given that limited number of studies and heterogeneity affect the reliability of the results.

### 4.2. Limitations

Although this review showed the effect of MI effects on pain in athletes with lower limb sports injuries, the limited number of RCT studies was taken into account as the limitation of this review. Further studies need to investigate the effect of MI by giving specific guidelines and using a homogeneous population of athletes in order to establish the efficacious effects in the rehabilitation process and in athletes’ ‘return to play’ period. Moreover, we tried to minimize the possibility of missing or unidentified publications with an extensive research method. However, the risk of biased selection towards positive results remains. As stated previously, we identified the methodological limitations of studies such as small sample sizes, different characteristics of the subjects, and different imagery modalities. Relaxation and Imagery was used in two of the three studies and therefore pain intensity could have been influenced. Somatic relaxation could have reduced muscle tension which is associated with pain intensity, thus affecting ΜΙ results. Additionally, the differential effects of MI perspectives (internal or external) could have influenced the results of the current study. The variety of mechanisms and cortical activations may change depending on the type and focus of the imagery, which means that is very difficult to draw any concrete conclusions about the influence on pain intensity. However, our analysis with respect to the limited number of studies, their methodological limitations and substantial heterogeneity may have affected the results of meta-analysis.

## 5. Conclusions

The findings of this systematic review indicate that between the 3 selected studies there were conflicting methodological procedures of MI implementation which could affect pain intensity in lower limb sports injuries. The meta-analysis showed no statistically significant positive effects of MI intervention on pain intensity after lower limb sports injuries, indicating no statistical difference and moderate to high heterogeneity. However, our findings indicate that the use of MI could reduce pain intensity compared to cases when it is not used. 

Finally, future studies could explore or investigate the effect of the type of MI interventions on pain intensity focusing on the different mechanisms and cortical activations in athletes with lower limb injuries.

## Figures and Tables

**Table 1:** Characteristics of the included studies.

Study	Age; *n*; Sex; Athletic Level;	Injury	Methods	Intervention	Results	PEDro Scale
1. Christakou and Zervas [44]	18–30 yrs; *n* = 18; M; at least 2 years of athletic experience	Ankle sprain grade II	Randomly allocated into 2 groups: 1. RI (*n* = 9), 2. CNTL, (*n* = 9), (Physical Therapy Program)	RI protocol 12 sessions/ 4 weeks 45 min each session	VAS: No significant statistical difference in the RI condition compared with the CNTL condition	7/11
2. Cupal and Brewer [45]	18–50 yrs; *n* = 30; M = 16, F = 14; recreational and competitive athletes	ACL reconstructive surgery	Randomly allocated into 3 groups: 1. RGI (*n* = 10), 2. PL (attention, encouragement and support), (n = 10), 3. CNTL Group (Physical Therapy Program)	RGI protocol 10 sessions every 2 weeks apart over 6 months/10–15 min (visual, kinesthetic, motivational and healing imagery)	NRS: Significant statistical difference in RGI condition compared with the PL and the CNTL condition	7/11
3. Lebon, Guillot, and Collet [46]	18–40 yrs; n = 12; M = 10, F = 2; recreational and competitive athletes	ACL reconstructive surgery	Randomly allocated into 2 groups: 1. KI (*n* = 7), 2. CNTL, (*n* = 5), (Physical Therapy Program)	KI protocol 12 sessions/5 weeks every second day/15 min each session	VAS: No significant statistical difference in the KI condition compared with the control condition	9/11

yrs: years, VAS: Visual Analogue Scale, NRS: Numerical Rating Scale, M: Male, F: Female, RI: Relaxation and Imagery, CNTL: Control, RGI: Relaxation and Guided Imagery, PL: Placebo, KI: Kinesthetic Imagery.

**Table 2:** PEDro Scale of Included Studies.

Study	Eligibility Criteria	Randomization	Allocation Concealed	Baseline Criteria	Participants Blinded	Therapist Blinded	Assessor Blinded	Minimum of 85% at Follow-Up	Statistical Analysis	Analysis between Groups	Point and Variance Data	Total
Christakou and Zervas [44]	Y	Y	Y	U	N	N	Y	U	Y	Y	Y	7/11
Cupal and Brewer [45]	Y	Y	N	Y	N	N	N	Y	Y	Y	Y	7/11
Lebon et al. [46]	Y	Y	Y	Y	N	N	Y	Y	Y	Y	Y	9/11

Y: Yes, N: No, U: Unclear.

**Table 3:** GRADE.

Certainty Assessment							No. of Patients		Effect	Certainty
No. of Studies	Study Design	Risk of Bias	Inconsistency	Indirectness	Imprecision	Other Considerations	Motor Imagery	Control	Absolute (95% CI)	
Christakou and Zervas [44] Cupal and Brewer [45] Lebon, Guillot and Collet [46]	randomized trials	not serious	serious ^b^	not serious	serious ^a^	none	26	24	MD −1.57 (−3.60 to 0.46)	⨁⨁◯◯ LOW

CI: Confidence interval; MD: mean difference; a: Sample < 400; b. Minimal or no overlap of CI; Heterogeneity (*p* < 0.05); I^2^ > 50%.

## Data Availability

Not applicable.
